# Advances in CMV Management: A Single Center Real-Life Experience

**DOI:** 10.3389/fcell.2020.534268

**Published:** 2020-10-27

**Authors:** Michele Malagola, Caterina Pollara, Nicola Polverelli, Tatiana Zollner, Daria Bettoni, Lisa Gandolfi, Doriana Gramegna, Enrico Morello, Alessandro Turra, Silvia Corbellini, Liana Signorini, Giovanni Moioli, Simona Bernardi, Camilla Zanaglio, Mirko Farina, Tullio Elia Testa, Arnaldo Caruso, Domenico Russo

**Affiliations:** ^1^Chair of Hematology, Bone Marrow Transplant Unit, ASST-Spedali Civili Brescia, Department of Clinical and Experimental Sciences, University of Brescia, Brescia, Italy; ^2^ASST-Spedali Civili, Section of Microbiology, Department of Experimental and Applied Medicine, University of Brescia, Brescia, Italy; ^3^UO Farmacia Aziendale, ASST Spedali Civili of Brescia, Brescia, Italy; ^4^Department of Infectious and Tropical Diseases, University of Brescia, ASST Spedali Civili di Brescia, Brescia, Italy; ^5^CREA Laboratory (Hematological-Research AIL Centre), ASST-Spedali Civili Brescia, Brescia, Italy

**Keywords:** CMV, allogeneic stem cell transplantation, prophylaxis, pre-emptive therapy, CMV DNA monitoring

## Abstract

CMV infection is a major challenge in allogeneic stem cell transplantation (allo-SCT). The changing landscape in CMV management includes the introduction of letermovir in prophylaxis of high-risk patients and the source of CMV DNA monitoring (plasma—PL vs. whole blood—WB), for pre-emptive therapy (PET) initiation. We report here how our real-life experience in CMV management evolved, following letermovir registration. We focus on: (i) the effects of systematic use of letermovir for CMV prophylaxis in high-risk patients, (ii) the results of a longitudinal comparison of CMV DNAemia monitoring in PL and WB. From December 2018 to April 2020, 60 allo-SCTs have been performed in our center (LET *ERA*), of whom 45 received letermovir in prophylaxis from day 0 to day + 100, because of recipient positivity of anti CMV IgG. These patients were compared with a cohort of 41 allo-SCTs performed between November 2017 and November 2018 (NO LET *ERA*). Firstly, the incidence of CMV clinically significant infections, CMV disease, bacterial infections, proven/probable fungal infections, hospital re-admissions after allo-SCT by day + 100 in the two ERA were 8 vs. 44% (*p* = 0.0006), 2 vs. 12% (*p* = 0.02), 37 vs. 56% (*p* = 0.05), 8 vs. 19% (*p* = 0.09), and 23 vs. 39% (*p* = 0.09), respectively. By day + 180 these differences were 17 vs. 68% (*p* < 0.00001), 2 vs. 12% (*p* = 0.02), 45 vs. 78% (*p* = 0.09), 8 vs. 22% (*p* = 0.05), and 40 vs. 66% (*p* = 0.01), respectively. Secondly, from February to May 2019, we comparatively measured CMV DNA from WB and PL and we confirmed that there is a linear correlation between CMV DNA level in WB and PL (Spearman’s test *r* = 0.86). Moreover, CMV DNAemia at the time of PET in the 12 patients with a clinically significant CMV infection was higher in WB vs. PL (5.202 vs. 4.981 copies/ml, *p* = 0.1). Our real-life experience confirms that: (i) letermovir is highly effective, leading to a significant drop in CMV clinically significant infections and CMV-related complications by day + 100 and + 180 after allo-SCT; (ii) WB may be an effective alternative to PL as a source for CMV DNA monitoring, as a linear correlation of DNAemia was confirmed between WB and PL, even if the CMV DNAemia at PET initiation was comparable in the two sources.

## Introduction

Cytomegalovirus (CMV) infection is a challenge in allogeneic stem cell transplantation (allo-SCT) (Boeckh and Nichols, 2004; Boeckh and Ljungman, 2009). In the first 100 days after transplant, it is detected in more than 60% of CMV seropositive recipients, in whom it produces a number of direct and indirect relevant effects, leading to an increase in non-relapse mortality (NRM). CMV disease, drug-related peripheral blood cytopenia, bacterial or fungal infections, and graft vs. host disease (GVHD) are the main CMV-related complications (Ariza-Heredia et al., 2014).

Before the availability of letermovir, the management of CMV infection was based on the monitoring of CMV DNAemia by RT-qPCR and on the prompt use of pre-emptive therapy (PET), either with foscarnet, ganciclovir, or valganciclovir. CMV DNA cut-off values for PET are still a matter of debate: Italian guidelines suggest more than 1,000 copies/ml in plasma (PL) or 10,000 copies/ml in whole blood (WB), in two consecutive assessments (Girmenia et al., 2019), while the ECIL-7 guidelines suggest that it should be adapted according to the monitoring technique used at the transplant center (Ljungman et al., 2019). PET is generally continued for at least 2 weeks, and stopped after at least one (Ljungman et al., 2019) or preferably two consecutive negative tests (Girmenia et al., 2019). Although anti-CMV hyper-immunoglobulins (Megalotect) can be safely used (Malagola et al., 2019), no conclusive data are available to recommend a routine use, and both the ECIL-7 guidelines and the Italian guidelines do not recommend the routine use of anti-CMV hyper-immunoglobulins (Girmenia et al., 2019; Ljungman et al., 2019).

Recently, significant improvements have been made in the management of CMV infection. They include: (i) the introduction of letermovir for prophylaxis in CMV seropositive patients; (ii) the advances in laboratory monitoring of CMV infection.

Letermovir has been licensed in Italy (December 2018) for prophylaxis in high-risk patients from day 0 to day + 100, following the conclusive data reported in the registration trial (Marty et al., 2017), showing a reduction of CMV clinically significant infection. In this study, the incidence of CMV reactivation was significantly lower in patients receiving letermovir vs. placebo (37 vs. 60%, respectively, *p* < 0.001), with an excellent safety profile (Marty et al., 2017).

Although ganciclovir and foscarnet were widely investigated in the 80s and 90s for CMV prophylaxis and have been proven to be effective in reducing CMV infection and disease, they showed significant toxicity (myelotoxicity for ganciclovir and nephrotoxicity for foscarnet), that hampered their extensive use in clinical practice (Chen et al., 2018). Thus, letermovir is, at present, the most effective and safe drug for CMV prophylaxis and the only licensed one for this indication.

During the last few years, the laboratory monitoring of CMV and the diagnosis of CMV infection moved from CMV antigenemia (Cariani et al., 2007) to molecular quantitative RT-qPCR (Girmenia et al., 2019; Ljungman et al., 2019), but a unique standardized method for detection of DNAemia has not been defined yet. Even though it is true that both WB and PL are valid sources for CMV DNA monitoring after allo-SCT, recently, WB has been suggested to be more reliable than PL as a source for RT-qPCR. In fact, in WB both intra-cellular and extra-cellular CMV DNA is detected, and during PET the clearance of CMV DNA appears to be faster in WB than in PL, where free particles of non-infective DNA are measured for many days after the complete clearance of the virus. This latter point is crucial, because it reduces the duration of PET and its side effects (Lazzarotto et al., 2018).

The aim of our study is to depict if and how letermovir and the availability of different sources for CMV DNAemia monitoring have impacted on daily CMV management. Thus, we report here a real-life single center experience in CMV management, conducted in our transplant center since letermovir registration in Italy (LET *ERA*) from December 2018 to April 2020, during which 60 allo-SCTs have been performed. We compared these data with a cohort of 41 patients allotransplanted before letermovir registration (from November 2017 to November 2018, NO LET *ERA*). We highlight two major issues: (i) the effects of the systematic use of letermovir for CMV prophylaxis in CMV positive recipients; (ii) the comparative monitoring of CMV DNAemia from WB and PL, to evaluate the reliability of the two methods in the era of prophylaxis with letermovir.

## Patients and Methods

### CMV Management Before Letermovir Introduction (Before December 2018)

For the purpose of this study, we report the policy and the results of CMV management before and after December 2018, that is when letermovir was licensed in Italy and routinely used by us for CMV prophylaxis in CMV seropositive patients undergoing allo-SCT.

Data were obtained by local databases and clinical charts, and special queries were addressed on missing data. The allo-SCT procedures, in terms of conditioning regimens, GVHD prophylaxis, and antimicrobial prophylaxis, were based on local guidelines and protocols, were undertaken upon written informed consent for transplant procedures and they remained unchanged following letermovir registration.

In order to define the risk of developing CMV infection and/or disease, standard practice included a serological test for CMV IgG of both the patient and the donor: patient’s CMV IgG positivity defined the high-risk category. Additional risk-factors were considered: the presence of a seronegative donor for a seropositive recipient, the presence of a mismatch donor, and the presence of GHVD, as reported in the literature (Styczynski, 2018). Up to December 2018, no prophylaxis against CMV was adopted, and all the patients received a standard-dose of acyclovir for other herpes virus prophylaxis. PET consisted of foscarnet, ganciclovir, or valganciclovir. Foscarnet was chosen in cases of peripheral blood cytopenia, ganciclovir in cases of renal impairment, and valganciclovir in cases of management of CMV once the patient has been discharged. PET was started in cases of a clinically significant CMV infection, defined as CMV reactivation with at least two positive controls (>1,000 copies/ml from PL) (Boeckh and Ljungman, 2009). From 2016, anti-CMV hyper-immunoglobulins (Megalotect) have been used in three settings: with anti-CMV specific drugs (during PET), in monotherapy for secondary prophylaxis (prevention of CMV breakthrough infection in very high-risk patients), and in order to control a CMV reactivation not requiring PET. Megalotect was used at the conventional dose of 100 UI/Kg every 2 weeks.

### CMV Management After Letermovir Introduction (After December 2018)

Following letermovir registration and availability in our hospital (January 2019), all high-risk patients were selected to receive standard prophylaxis with letermovir from day 0 to day + 100, at the conventional dose of 240 or 480 mg/day orally, based on the immunosuppressive drug co-administered for GVHD prophylaxis. No changes were made in PET approach, as well as in the supportive care with Megalotect. In particular, we maintained the clinical practice to start PET following two consecutive CMV positive samples with more than 1000 copies/ml in PL. Nevertheless, soon after the introduction of letermovir (from February 2019 to May 2019), we conducted a comparative assessment of CMV-DNA from PL and WB, in order to verify WB reliability, with the intent to move from PL to WB, as a source for CMV DNA monitoring (from June 2019). During this time-frame, any decision for PET initiation was made considering the CMV-DNA on PL. After May 2019, a cut-off of 10,000 copies/ml in two consecutive samples was adopted for PET initiation. Moreover, for DNAemia between 1000 and 10,000 copies/ml the presence of additional risk factors for CMV-related complications was carefully considered for a prompt PET start, even with copies < 10,000/ml in two consecutive samples (e.g., aGVHD under steroids and haploidentical donor).

### CMV DNAemia Monitoring

Up to February 2019, the clinical practice in our transplant center was to monitor CMV-DNA from PL at least once a week until day + 100, and to start PET in cases of clinical significant CMV infection as above reported. From February to May 2019, in collaboration with the microbiology unit of our hospital, we carried out a CMV DNAemia monitoring protocol for allotransplanted patients for comparative determination of quantitative CMV DNA from WB and PL. In particular, this comparative evaluation was performed both in patients who were transplanted between February and March 2019 (*n* = 21) and in patients who have been previously transplanted and were monitored during the follow up and transplanted before letermovir availability (*n* = 47). Extraction, detection, and quantification of CMV-DNA in paired WB and PL samples were performed using a commercial automated platform (ELITe InGenius^®^, Elitechgroup, Italy) (CMV ELITe MGB^®^ Kit, 2017, SCH mRTK015PLD_11, of 10/03/17, Elitechgroup). The ELITe InGenius^®^ instrument is the first fully automated sample-to-result solution, integrating sample preparation, amplification, and result analysis, validated with a quantitative transplant pathogen monitoring menu, based on the real-time PCR MGB technology. Results interpretation and analysis are automatically done by the ELITe InGenius^®^ system. In comparison to conventional PCR platforms and conventional methods in microbiology, the ELITe InGenius^®^ system simplified and reduced the duration of the pre-analytical and analytical phases in the laboratory (2 h 30’ to process one sample) and proved to be a reliable and sensitive tool for a sample-in answer-out detection of CMV directly from clinical samples.

Briefly, primary samples were loaded directly and processed in the ELITe InGenius^®^ system, according to the manufacturer’s instructions. The instrument collected only 200 μL for each sample (WB and PL) and the purified nucleic acid was eluted into a total volume of 100 μL and amplification was performed using 20 μL. For each protocol, the limit of detection (LoD) and the lower limit of quantification (LLoQ) were as reported by the manufacturer. In particular, the LoD was 156 and 293 copies/ml from WB and PL, respectively, and the LLoQ was 254 and 593 copies/ml from WB and PL, respectively. Quantitative results were reported as log10 copies/mL of the sample. Positive samples (WB and PL) below the LLoQ were considered negative; while all samples with a value < 156 copies/mL for WB and < 293 copies/ml for PL were considered really negative (0 copies/mL) samples. For the ELITe InGenius^®^ real-time CMV assay, the manufacturer has determined the conversion factor and provides a conversion factor whatever the matrix used (WB or plasma). The conversion factor for WB is 2.9 IU/copies, while for PL is 1.9 IU/copies.

### Statistical Analysis

CMV DNA levels between whole blood and plasma samples were compared by a Mann-Whitney *U*-test. The correlation between CMV DNA in the two groups was analyzed using the Spearman’s correlation coefficient (Dioverti et al., 2017). The differences between the groups were analyzed using the Chi-square test or the Fisher’s exact test, as appropriate. A *P-*value of < 0.05 was considered as statistically significant.

## Results

### Results of Letermovir Prophylaxis

For the purpose of this study, we consider 110 allogeneic transplants performed from November 2017 to April 2020 in our transplant center, divided into two cohorts: 41 transplants performed before the registration of letermovir in Italy (NO LET *ERA*—November 2017–November 2018) and 60 transplants performed after letermovir registration in Italy (LET *ERA*—December 2018–April 2020).

Table 1 reports the clinical and transplant characteristics of the two cohorts. Focusing on the 60 transplants of the LET *ERA*, more than 50% were performed for acute leukemia, 53% were made in complete remission, and 57% following a myeloablative conditioning regimen. In 70% of them peripheral blood stem cells were used, and 27% were performed with a haploidentical transplantation. No statistical significant differences were observed in the two cohorts, with the exception of the expected difference in letermovir use: letermovir prophylaxis was used in 45/60 transplants of the LET *ERA* (75%) and in none of the transplant of the previous period (*p* < 0.0001). All the patients who received letermovir were IgG positive. Additional CMV risk factors were: matched unrelated donor (23 cases) and haploidentical donor (14 cases). Most of the patients (80%) who received letermovir prophylaxis took 240 mg/daily together with cyclosporine. Nine patients (20%) received letermovir 480 mg together with sirolimus (6 cases) or tacrolimus (3 cases). At the last follow up (April 20, 2020), 25/45 patients (56%) discontinued letermovir because they completed the planned 100 days of treatment, while 13/45 patients (29%) were still receiving letermovir and 7/45 (15%) discontinued letermovir for CMV clinically significant infection or death before day + 100.

**TABLE 1:** Clinical and transplant characteristics of the 101 patients transplanted from November 2017 to April 2020.

**Characteristics**	**NO LETERMOVIR *ERA***		**LETERMOVIR *ERA***		
	**Nov 2017–Nov 2018**		**Dec 2018–Apr 2020**		**P**
	***N* = 41**		***N* = 60**		
	**N**	**%**	**N**	**%**	
Patient age, median (range)	56 (19–71)	–	52 (21–71)	–	
Patient sex, female/male	19/22	46/54	27/33	45/55	0.89
**Disease**					
AL	27	66	32	53	0.20
MFI	4	10	9	15	0.43
MM	4	1014	8	13	0.58
NHL	6	0	5	8	0.31
HL	0	0	2	3	n.e.
SAA	0		2	3	n.e.
Other			2	3	n.e.
**Disease status at transplant**					
CR	21	51	32	53	0.83
CMV serostatus (R +)	32	78	51	85	0.37
**Donor type**					
Sibling	11	27	11	18	0.07
MUD	24	58	32	53	0.69
Haplo	15	15	16	27	0.38
UCB	0	0	1	2	1
**Stem cell source**					
PBSC	34	83	42	70	0.16
BM	7	17	17	28	0.24
UCB	0	0	1	2	1
**Conditioning intensity**					
RIC	21	51	26	43	0.54
MAC	20	49	34	57	
aGVHD grade II-IV	12	29	14	23	0.64
cGVHD	3	7	2	3	0.39
**Letermovir**					
YES	0	0	45	45	< 0.0001
NO	41	100	15	15	
**Letermovir duration**					
Discontinued at day + 100	–	–	25	56	–
Ongoing	–	–	13	29	–
Discontinued before day + 100	–	–	7	15	–
**Dose**					
240 mg/day	–	–	36	80	–
480 mg/day	–	–	9	20	–

*AL, acute leukemia; MFI, primary myelofibrosis; HL, Hodgkin lymphoma; NHL, Non-Hodgkin lymphoma; MM, multiple myeloma; CR, complete remission; MUD, matched unrelated donor; Haplo, haploidentical; UCB, umbilical cord blood; PBSC, peripheral blood stem cell; BM, bone marrow; MAC, myeloablative conditioning; RIC, reduced intensity conditioning; aGVHD, acute graft vs. host disease; cGVHD, chronic graft vs. host disease; n.e., not evaluable.*

Overall, the treatment was very well tolerated. In particular, no drug-related grade ł 2 WHO adverse events were reported (diarrhea, vomiting, skin rash, fever, cough, or peripheral edema) (Marty et al., 2017).

As above reported, considering the 60 transplants of the LET *ERA* letermovir prophylaxis was used in 45 cases (75%, Table 2a). In total, 14/45 (31%) developed a CMV reactivation (before day + 100, during letermovir prophylaxis in 8 cases and after day + 100 in 6 cases). In 7 cases (7/45, 15%) the infection was clinically significant and required PET (2 cases—4%—during letermovir prophylaxis, before day + 100 and 5 cases—11%—after day + 100).

**TABLE 2a:** CMV course in the 45 high-risk (CMV IgG+) patients who received prophylaxis with letermovir from day 0 to day + 100.

**Pt N°**	**SCT date**	**CMV IgG D/R**	**Day of 1st CMV reactivation**	**CMV DNAemia**	**Source**	**PET**	**LET duration**	**Pt last f up**
1	Mar 2020	−/+	+10	<1,000 copies/ml	WB	–	27	+27
2	Jul 2019	+/+	+11	<1,000 copies/ml	WB	–	100	+234
3	Oct 2019	+/+	+11	<1,000 copies/ml	WB	–	82	+192
4	Jan 2019	+/+	+12	2,779 copies/ml	PL	FOS	12	Dead
5*	Mar 2019	−/+	+13	10,099 copies/ml	PL	FOS	13	Dead
6	May 2019	+/+	+20	<1,000 copies/ml	WB	–	100	Dead
7	May 2019	−/+	+20	<1,000 copies/ml	WB	–	100	+343
8	Jan 2019	−/+	+78	<1,000 copies/ml	PL	–	100	+493
9	Feb 2019	+/+	+120	2,280 copies/ml	PL	VAL	100	+433
10	Feb 2019	+/+	+130	<1,000 copies/ml	WB	–	100	+443
11	Mar 2019	−/+	+133	10,744 copies/ml	WB	VAL	100	+383
12	Oct 2019	+/+	+146	10,900 copies/ml	WB	VAL	100	+186
13	May 2019	+/+	+147	4,675 copies/ml	WB	VAL	100	+332
14	Jul 2019	+/+	+150	5,451 copies/ml	WB	VAL	100	+178
15	Jan 2019	+/+	–	–	–	–	100	Dead
16	Jun 2019	+/+	–	–	–	–	18	Dead
17	Jun 2019	+/+	–	–	–	–	68	Dead
18	Jun 2019	+/+	–	–	–	–	100	+307
19	Jul 2019	+/+	–	–	–	–	100	+292
20	Jul 2019	−/+	–	–	–	–	100	+283
21	Jul 2019	−/+	–	–	–	–	100	+270
22	Sep 2019	+/+	–	–	–	–	100	+214
23	Oct 2019	+/+	–	–	–	–	100	+193
24	Oct 2019	−/+	–	–	–	–	100	+116
25**	Nov 2019	−/+	–	–	–	–	40	Dead
26	Nov 2019	−/+	–	–	–	–	100	+156
27	Nov 2019	+/+	–	–	–	–	100	+155
28	Nov 2019	+/+	–	–	–	–	24	Dead
29	Nov 2019	+/+	–	–	–	–	100	+143
30	Dec 2019	−/+	–	–	–	–	100	+129
31	Dec 2019	−/+	–	–	–	–	100	+122
33	Jan 2020	−/+	–	–	–	–	100	+102
33	Jan 2020	+/+	–	–	–	–	100	+101
34	Jan 2020	+/+	–	–	–	–	95	+95
35	Jan 2020	+/+	–	–	–	–	80	+80
36	Feb 2020	+/+	–	–	–	–	20	Dead
37	Feb 2020	−/+	–	–	–	–	66	+66
38	Feb 2020	+/+	–	–	–	–	60	+60
39	Feb 2020	+/+	–	–	–	–	56	+56
40	Mar 2020	+/+	–	–	–	–	41	+41
41	Mar 2020	+/+	–	–	–	–	25	+25
42	Apr 2020	+/+	–	–	–	–	11	+11
43	Apr 2020	+/+	–	–	–	–	10	+10
44	Apr 2020	−/+	–	–	–	–	6	+6
45	Apr 2020	+/+	–	–	–	–	3	+3

*First transplant for primary myelofibrosis.

**First transplant for primary myelofibrosis. Letermovir discontinuation at day + 40 because of foscarnet therapy for HHV6 reactivation.

A total of 15/60 (25%) patients of the LET ERA did not receive letermovir prophylaxis (CMV seronegativity in 9 cases, letermovir temporary unavailability at our institution in 3 cases, second transplant in 2 cases, who previously received letermovir for the first transplant and received an anti-CMV drug during the follow up and CMV DNA positivity at day −2 in 1 case; Table 2b). In total, 5/15 of these patients (45%) developed a CMV-related complication: two patients developed a clinically significant CMV infection at day +13 and +24, one at day −2 and two patients developed a CMV disease (1 lung localization before day + 100 and 1 gut localization after day + 100, both without CMV DNAemia).

**TABLE 2b:** CMV course in the 15 patients who did not receive prophylaxis with letermovir from day 0 to day + 100.

**Pt ID**	**SCT date**	**CMV IgG D/R**	**Day of 1st CMV** **reactivation**	**CMV DNAemia**	**Source**	**Anti CMV therapy**	**Pts last f up**
46*	Feb 2019	−/+	−2	2,605 copies/ml	PL	FOS	+421
47	Apr 2019	−/−	+13	1,347 copies/ml	PL	VAL	+376
48	Mar 2019	+/−	+20	–	–	FOS^#^	Dead
49^@^	Dec 2018	−/+	+24	2,273 copies/ml	PL	FOS	+485
50^@^	Dec 2018	−/+	+240	–	–	GAN^§^	+478
51	Dec 2018	−/+	–	–	–	–	Dead
56	Apr 2019	–/–	–	–	–	–	+335
5**	Apr 2019	–/+	–	–		–	Dead
57	Jun 2019	–/–	–	–		–	+294
58	Aug 2019	−/−	–	–		–	+260
25***	Dec 2019	−/+	–	–		–	Dead

*Letermovir was not used because of CMV positive DNA before SCT (day −2).^#^FOS for CMV disease (lung) on day + 16, with negative CMV DNA on PL.^@^Letermovir was not used because, although registered, it became available in our hospital from January 2019.^§^ GAN for CMV disease (gut) on day + with negative CMV DNA on PL.**Letermovir was not used because this is a second transplant. Letermovir was used in the first transplant and discontinued at day + 13 for CMV clinically significant infection (see Table 2a).***Letermovir was discontinued at day + 40 after the first transplant because of foscarnet therapy for HHV6 reactivation (see Table 2a).

Considering all the 12 cases of CMV clinically significant infection or disease observed in the LET *ERA* (7 in the letermovir group—Table 2a—and 5 in the non-letermovir group—Table 2b), the treatment consisted of foscarnet (5 cases), valganciclovir (6 cases), and ganciclovir (1 case). All the patients who received PET for a clinically significant CMV infection achieved a complete clearance of CMV DNA after a median of 14 days (range 7–26). In 5/12 cases (42%) a breakthrough CMV infection was observed, that was successfully treated with second line anti-CMV drugs. No resistance to ganciclovir or foscarnet was observed.

In order to assess the impact of letermovir prophylaxis in the clinical management of allotransplanted patients, we compared the most relevant transplant outcomes in the NO LET *ERA* (Nov 2017–Nov 2018; 41 transplants) vs. the LET *ERA* (Dec 2018–Apr 2020; 60 transplants) (Table 3). Focusing on the first 100 days after allo-SCT, the incidence of clinically significant CMV infections, CMV disease, bacterial infections, proven/probable fungal infections, and hospital readmission was 44 vs. 5% (*p* = 0.0006), 12 vs. 2% (*p* = 0.02), 56 vs. 37% (*p* = 0.05), 19 vs. 8% (*p* = 0.09%), and 39 vs. 23% (*p* = 0.09), respectively. Moreover, the incidence of grade ł 2 aGVHD was 29 vs. 23%, in the two periods (*p* = 0.5). The observed differences were more evident prolonging the observation up to 180 days after allo-SCT: 68 vs. 17% for CMV clinically significant infection (*p* < 0.00001), 12 vs. 2% for CMV disease (*p* = 0.02), 78 vs. 45% for bacterial infections (*p* = 0.009), 22 vs. 8% for proven/probable fungal infections (*p* = 0.05), and 66 vs. 40% for hospital re-admission (*p* = 0.01). The costs of PET showed a reduction from Euro 38,000 to Euro 10,000, that only partially balanced the costs of letermovir from day 0 to day + 100 (Euro 13,700/pt for 240 mg/daily and Euro 29,000/pt for 480 mg/daily).

**TABLE 3:** Impact of letermovir on the management of patients undergoing allo-SCT.

	**Nov 2017–Nov 2018**	**Dec 2018–APR 2020**	***P***
	**NO LET *“ERA”***	**LET *“ERA”***	
No. of allo-SCT	41	60	–
Letermovir prophylaxys day 0 → + 100	0	45	–
Clinically significant CMV infection (≤ 100 days)	18 (44%)	5* (8%)	0.0006
Clinically significant CMV infection (≤ 180 days)	28 (68%)	10 (17%)	<0.00001
CMV disease (≤100 days)	5 (12%)	1** (2%)	0.02
CMV disease (≤180 days)	5 (12%)	1 (2%)	0.02
Bacterial infections (≤100 days)	23 (56%)	22 (37%)	0.05
Bacterial infections (≤180 days)	32 (78%)	27 (45%)	0.009
Fungal infections (probable/proven) (≤100 days)	8 (19%)	5 (8%)	0.09
Fungal infections (probable/proven) (≤180 days)	9 (22%)	5 (8%)	0.05
aGVHD grade ≥ 2	12 (29%)	14 (23%)	0.5
Hospital re-admission (≤100 days)	16 (39%)	14 (23%)	0.09
Hospital re-admission (≤180 days)	27 (66%)	24 (40%)	0.01
Cumulative cost for PET^#^	Euro 38,000	Euro 10,000	–
Letermovir costs			
−240 mg day 0 → + 100	–	Euro 13,700/pt	–
−480 mg day 0 → + 100	–	Euro 29,000/pt	–

*Two patients in the letermovir group (CE and CP in Table 2a) and 3 patients in the no-letermovir group (CN, BG, and FB in Table 2b).**Two patients in the no-letermovir group (BA and FG in Table 2b).^#^This indicates the cumulative cost with the different anti-CMV specific drug actually administered (FOS, GAN, VAL), calculated for 15 days and for a standard of 70 Kg of body weight.

### Comparative Monitoring of CMV DNAemia From PL and WB

From February to May 2019 (4 months), a total of 566 consecutive samples were collected, for comparative assessment of CMV DNA from PL and WB. The samples came from 68 patients (21 females and 47 males, mean age 50 ± 21 years), of whom 21 allotransplanted in the same period of time (250 samples) and 47 allotransplanted before February 2019 and who were monitored during follow up (316 samples). The median number of samples per patient was 8 (range: 1–29).

**FIGURE 1:**
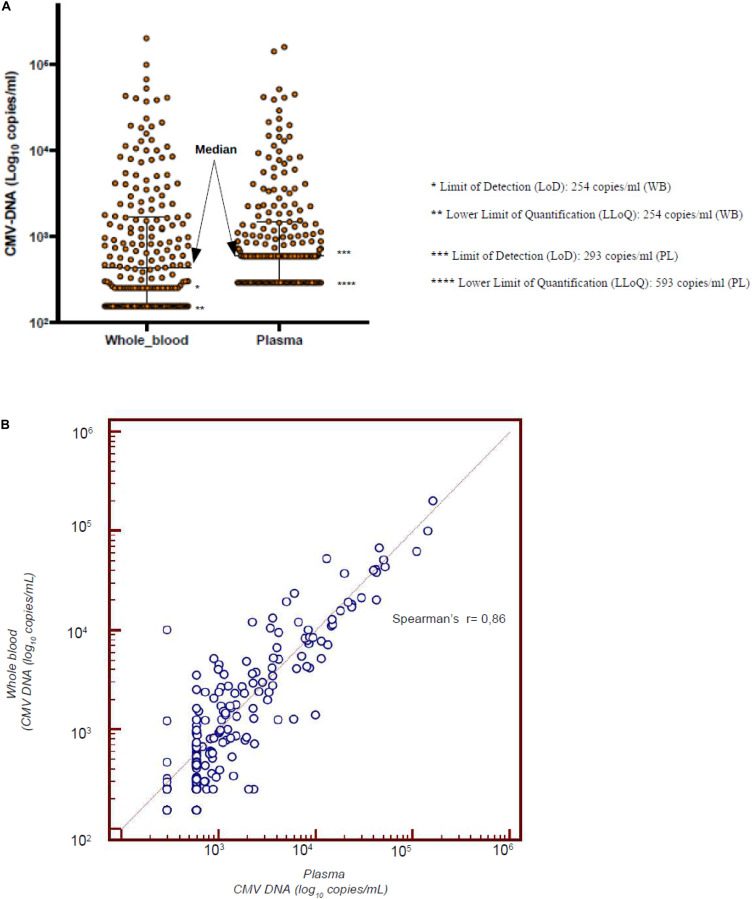
**(A)** Median value of CMV-DNAemia of the 18 patients with CMV reactivation who were monitored simultaneously from PL and WB. **(B)** Linear correlation between the CMV DNAemia assessed on PL and WB in the 18 patients who experienced a CMV reactivation.

The first objective of this analysis was to confirm the data reported in the literature of a linear correlation between the results of the RT-qPCR on PL and WB (Lazzarotto et al., 2018). Considering all the 566 samples, including 294 samples with undetectable CMV DNAemia, the median number of copies/ml was 293 (range 293–169,769) and 156 (range 156–202,261) on PL and WB, respectively. The Spearman’s test clearly showed a linear correlation between the two sources (*r* = 0.87). Considering the 272 samples coming from the 18 patients who had a CMV reactivation, the median value of CMV DNA was 593 copies/ml on PL and 402 copies/mL on WB, respectively (Figure 1A). The mean was 4,664 copies/ml on PL and 4,679 copies/mL on WB, respectively (Figure 1A). Figure 1B confirms the linear correlation between the values (*r* = 0.86).

**FIGURE 2:**
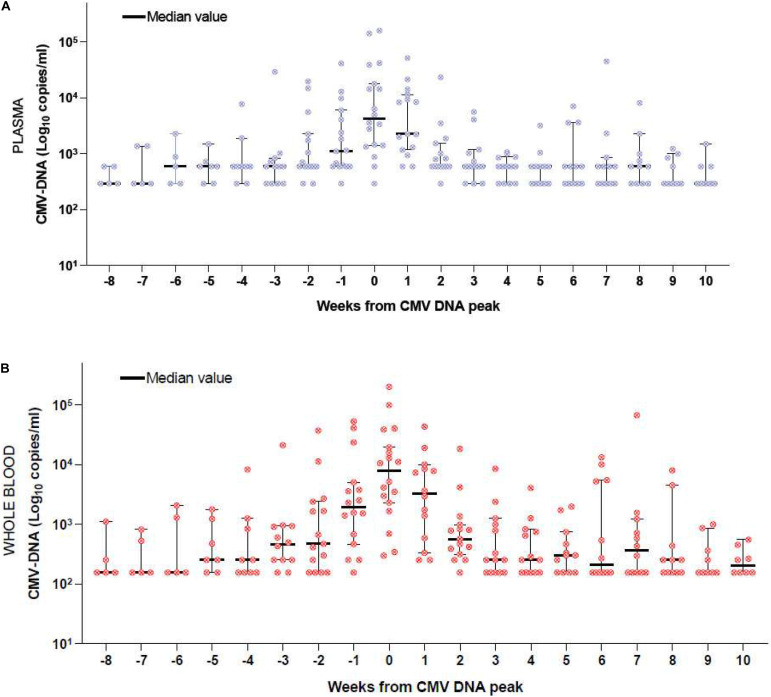
**(A)** CMV DNAemia monitored on PL of the 12 patients who received PET. Week 0 includes the maximum DNAemia. Negative weeks include the DNAemia before the peak and positive weeks include the DNAemia during PET. **(B)** CMV DNAemia monitored on WB of the 12 patients who received PET. Week 0 includes the maximum DNAemia. Negative weeks include the DNAemia before the peak and positive weeks include the DNAemia during PET.

We then grouped the CMV DNA level as detected from PL and WB of the 208 samples from the 12/18 patients who experienced a CMV reactivation that required PET. Figures 2A,B report this analysis. Time-point zero of each figure includes the peak value of CMV DNA which correspond to the first day of anti-CMV treatment; negative time-points include the weekly assessment before PET initiation and positive timepoints include the weekly assessment during PET. At time-point zero, the DNAemia on WB (Figure 2B, median 5,202 copies/mL) was higher than that detected on PL (Figure 2A, median 4,981 copies/mL). The difference was not significant (*p* = 0.1). The kinetics of CMV DNAemia during PET was similar when considering PL (Figure 2A) or WB (Figure 2B). The comparative CMV DNAemia expressed in copies/ml and IU/ml according to the manufacturer conversion factor is reported in Supplementary Table 1.

## Discussion

CMV management is a major challenge for patients admitted for allo-SCT and a raise in CMV reactivation is expected in the next few years following the extensive use of haploidentical transplantation and the progressive increase of patients’ age at transplant (Boeckh and Ljungman, 2009). In both these settings, acute and chronic GVHD incidence is expected to be higher in comparison to conventional transplants in younger patients. Thus the prolonged immunosuppression and the long-lasting immune-reconstitution are expecting to expose these patients to more frequent CMV infections.

The attention of the transplant community on CMV is particularly high, and recently the Gruppo Italiano Trapianto di Midollo Osseo (GITMO) published a guideline addressing the major points of CMV management (Girmenia et al., 2019). Nevertheless, the real-life policy of CMV management is still heterogeneous in this transition period, considering that letermovir has been recently registered for CMV prophylaxis (Marty et al., 2017) and that CMV DNA monitoring from WB instead of PL for definition of the CMV clinically significant infection has been proposed to be preferable, even though not definitively demonstrated and largely applied (Lazzarotto et al., 2018).

These clinical and laboratory aspects have been recently highlighted by Marty et al. (2017) in their registration studies and by Lazzarotto et al. (2018) in their experimental work. The results of both papers are considered as reference, but few data on real-life experience are currently available, taking into account that the number of patients who received primary prophylaxis with letermovir in the real-life published studies is very limited (Lin et al., 2019; Anderson et al., 2020; Johnsrud et al., 2020; Sharma et al., 2020).

In the present paper, we report a real-life experience on CMV management in our transplant center following letermovir registration in Italy (December 2018) and we compare a cohort of 60 transplants performed after letermovir registration (LET *ERA*, December 2018–April 2020) with a historical cohort of transplants performed before letermovir registration (NO LET *ERA*, November 2017–November 2018), with the aim to evaluate the efficacy of letermovir prophylaxis and its impact on CMV reactivation and incidence of clinically significant CMV infections. The results of our experience confirms that letermovir is highly effective and safe in reducing the incidence of CMV reactivation, when used from day 0 to day + 100. Impressively, the incidence of clinically significant CMV infection by day + 100 in the LET *ERA* was 8%, significantly lower than the 44% observed in the NO LET *ERA* (Table 3, *p* = 0.0006), even if, as reported in Table 2a, 8/45 patients (18%) and 11/45 patients (24%) have not completed the first 60 and 90 days of prophylaxis, respectively. The effectiveness of letermovir in reducing CMV infections and CMV-related events is even more evident if we prolong the observation up to day + 180 after allo-SCT. Our data are in line with the observations of Johnsrud and colleagues, as we found that by day + 180 the incidence of CMV clinically significant CMV infection, CMV disease, bacterial infection, probable/proven fungal infections, and the incidence of hospital re-admission reduced from 68, 12, 78, 22, and 66% to 17, 2, 45, 8, and 40%, respectively (*p* < 0.00001, *p* = 0.02, *p* = 0.009, *p* = 0.05, and *p* = 0.01, respectively; Table 3).

Facing the costs of PET before and after letermovir introduction, we observed a reduction in PET cost/year from Euro 38,000 to Euro 10,000, that was partially balanced by letermovir costs (Euro 13,700/pt at the dose of 240 mg/daily and Euro 29,000/pt at the dose of 480 mg/daily for 100 days). However, the cost-effectiveness should be calculated not only on the basis of the cost of drugs, but also considering the cost of hospital readmission, antibiotics, antifungal drugs, supportive therapy, and their impact on quality of life (Restelli et al., 2019).

Concerning the safety profile we confirm that the drug is highly safe, with neither hematological nor non-hematological adverse events. The drug was used at the dose reported in the investigator’s brochure: 240 mg/daily in 36 patients treated with cyclosporine and 480 mg/daily in 9 patients treated with other immunosuppressive drugs. No cumulative drug-related adverse events were recorded, the drug was used from day 0 in all the patients, and no detrimental effects on engraftment and/or negative effects on the frequency and severity of GVHD were observed.

According to the registration indication, letermovir was discontinued by day + 100, but the patients continued to be monitored and 5/45 (11%) additional patients had late-onset new CMV reactivation at the last follow up (Table 2a). This event is of interest, and a higher number of late CMV-related events (infections and disease) are expected to be registered in the next few years, following the extensive use of haploidentical transplantation and the increasing age of patients. Thus, we think that letermovir may be useful also after day + 100 too and, in order to clearly demonstrate this, we need to wait the results of the randomized phase III multicentric trial exploring letermovir vs. placebo from day + 100 to day + 200, in which we are currently recruiting patients in our center (Clinicaltrials.gov: NCT03930615).

The need to harmonize CMV management is crucial, in order to make data comparable between different centers. This harmonization also includes the specimen for CMV monitoring (PL or WB) and the cut-off for PET initiation. The pivotal paper by Lazzarotto and colleagues suggested that, at infection onset, CMV DNA in WB is usually higher than in PL (approximately 1 log) and that the kinetic of CMV DNA clearance during PET is more effectively depicted when WB is used, with a significant reduction in the number of days on PET (Lazzarotto et al., 2018). In our cohort of 12 patients who developed CMV reactivation and received PET between February and May 2019, we observed that the peak of CMV DNA on WB displayed an insignificantly higher number copies of DNA/ml vs. PL (5.502 vs. 4.891 copies/ml) (Figures 2A,B). Moreover, the kinetic of CMV DNA clearance during PET was comparable in the two sources (Figures 2A,B). We can speculate that this finding could be related to the small number of patients or could depend on technical reasons. Our system (ELITe InGenius^®^) is completely automatic and thus can optimally reduce the bias eventually associated with manual manipulation of blood samples. The next step will be the expression of CMV DNA in IU instead of copies/ml, by applying a conversion factor, with the aim to abolish any inter-laboratory variation (Sidoti et al., 2019). This process is ongoing in our center and in the GITMO scientific society, in the context of a multicentric study. From June 2019 we definitively moved from PL to WB and adopted 10,000 copies/ml in two consecutive samples as the threshold for PET initiation. When the DNAemia is > 1,000 copies/ml, but < 10,000 copies/ml, we usually perform a twice weekly longitudinal monitoring of CMV DNA, in accordance with the recently published guidelines, considering additional risk factors (e.g., GHVD, mismatch transplant, early infection, and alternative donor transplantation) (Girmenia et al., 2019). This is the case of patients 13 and 14 reported in Table 2a, who were treated with PET given a CMV DNAemia of 4,675 and 5,451 copies/ml, respectively, because of aGVHD under steroids in both cases.

## Conclusion

In conclusion, our real-life experience, moving from NO LET *ERA* to LET *ERA*, confirms the high efficacy and the safety of letermovir for CMV prophylaxis from day 0 to day + 100. The use of letermovir led to a significant reduction of CMV-indirect effects, such as bacterial infections, fungal infections, and hospital readmission by day + 180. We confirmed the data of the linear correlation between CMV DNA assessed in PL and WB, but we were not able to confirm two other observations reported in the literature (Lazzarotto et al., 2018): the 1-log higher DNAemia in WB vs. PL at the time of PET initiation and the more rapid reduction in CMV DNA during PET when WB is used. Thus, we suggest that each laboratory should perform internal analysis before moving definitively from PL to WB and we consider it good clinical practice to assess two or three consecutive RT-qPCR showing at least 0.5 Log of DNAemia increase as a sign of a clinically significant CMV reactivation which requires PET.

## Data Availability Statement

The datasets generated for this study are available on request to the corresponding author.

## Author Contributions

MM, CP, NP, AC, and DR designed the study. MM, TZ, LG, DG, EM, NP, AT, LS, GM, SB, CZ, and MF collected and analyzed the data. CP, SC, and AC performed the laboratory analysis form CMV DNA monitoring. DB and TT performed the costs analysis. MM, CP, and DR wrote the manuscript. All authors gave their final approval before submission.

## Conflict of Interest

MM was included in Biotest Advisory Board. DR was included in Merck Sharp and Dohme Advisory Board. The remaining authors declare that the research was conducted in the absence of any commercial or financial relationships that could be construed as a potential conflict of interest.
