# Supplementary material for: Advances in CMV Management: A Single Center Real-Life Experience
Source: Front Cell Dev Biol. 2020 Oct 27;8:534268. doi: 10.3389/fcell.2020.534268 (PMC7652755; doi:10.3389/fcell.2020.534268)
Supplement: Supplementary file 1 [file Table_1.doc]

**Supplementary Table. Comparison of the median DNA levels as expressed in copies/ml and IU/ml in PL and WB of the 12 patients who experienced a CMV clinically significant infections and received PET.** Week 0 is the maximum DNAemia; negative weeks refer to the evaluations before the peak; positive weeks refer to the evaluations during PET (See figure 2A and 2B)

| **Week** | **PL** (conversion factor 1,9 IU/copies) | | **WB** (conversion factor 2,9 IU/copies) | |
| --- | --- | --- | --- | --- |
|  | **Copies/ml** | **IU/ml** | **Copies/ml** | **IU/ml** |
| **-8** | 293 | 556.7 | 156 | 452.4 |
| **-7** | 293 | 556.7 | 156 | 452.4 |
| **-6** | 593 | 1126.7 | 156 | 452.4 |
| **-5** | 593 | 1126.7 | 254 | 736.6 |
| **-4** | 593 | 1126.7 | 254 | 736.6 |
| **-3** | 593 | 1126.7 | 603 | 1748.7 |
| **-2** | 1054 | 2002.6 | 1649 | 4782.1 |
| **-1** | 2124 | 4035.6 | 3072 | 8908.8 |
| **0** | 14532 | 27610,8‬ | 14395.5 | 417469,5 |
| **+1** | 8413.5 | 159856,5 | 7359 | 21863,1‬ |
| **+2** | 802 | 1523.8 | 787 | 2282,3 |
| **+3** | 593 | 1126.7 | 291 | 843,9 |
| **+4** | 593 | 1126.7 | 270.5 | 784,45‬ |
| **+5** | 593 | 1126.7 | 334 | 968,6 |
| **+6** | 593 | 1126.7 | 156 | 452.4 |
| **+7** | 593 | 1126.7 | 584 | 1.693,6 |
| **+8** | 593 | 1126.7 | 254 | 736.6 |
| **+9** | 443 | 841.7 | 205 | 594,5 |
| **+10** | 293 | 556.7 | 254 | 736.6 |
